# 1277. Colonization Rates for Antimicrobial-resistant Bacteria in Kenya: An Antibiotic Resistance in Communities and Hospitals (ARCH) Study

**DOI:** 10.1093/ofid/ofab466.1469

**Published:** 2021-12-04

**Authors:** Sylvia Omulo, Ulzii-Orshikh Luvsansharav, Teresa Ita, Robert Mugoh, Mark Caudell, Brooke M Ramay, Guy H Palmer, Linus Ndegwa, Jennifer Verani, Susan Bollinger, Aditya Sharma, Douglas Call, Rachel Smith

**Affiliations:** 1 Washington State University, Pullman, Washington; 2 CDC, Decatur, Georgia; 3 Washington State University Global Health - Kenya, Nairobi, Nairobi Area, Kenya; 4 Food and Agriculture Organization of the United Nations, Nairobi, Nairobi Area, Kenya; 5 Universidad del Valle de Guatemala, Center for Health Studies, Paul G. Allen School for Global Health, Washington State University, Pullman, USA, Guatemala City, Sacatepequez, Guatemala; 6 US CDC Kenya Office, Nairobi, Nairobi Area, Kenya; 7 Centers for Disease Control and Prevention, Atlanta, Georgia; 8 U.S. Centers for Disease Control and Prevention, Atlanta, Georgia

## Abstract

**Background:**

Characterization of antimicrobial-resistant organism (ARO) colonization is critical to understand transmission dynamics and infection risk, however data in resource-limited settings are scare. We estimated the prevalence of Enterobacterales colonization with extended-spectrum cephalosporin-resistance (ESCrE), carbapenem-resistance (CRE) and methicillin-resistant *Staphylococcus aureus* (MRSA) among community residents and hospitalized patients in rural (Siaya County) and urban (Kibera) Kenya.

**Methods:**

Community-dwelling adults and children were enrolled via cluster randomized sampling. Inpatients of all ages were enrolled by simple random sampling. Stool/rectal and nasal swabs were collected and screened for ESCrE, CRE and MRSA, respectively, using HardyChrom™ media. Vitek2^®^ was used for isolate confirmation and antibiotic susceptibility testing. Fisher’s exact tests were used to compare prevalence of AROs.

**Results:**

The prevalence of ESCrE was higher for the urban hospital (69.8%, 263/377) compared to rural hospitals (62.7%, 298/475, *P*=0.04); a similar pattern was evident for CRE (16.7%, 63/377 and 6.5%, 31/475, respectively, *P*< 0.01). The prevalence of MRSA was 3.2% for both urban and rural hospitals (*P*=0.99). For adults, the prevalence of ESCrE was higher in Kibera households (51.4%, 346/673) compared to Siaya (44.6%, 283/634, *P*=0.02) while the prevalence of both CRE and MRSA was < 3% for both areas and did not differ significantly (CRE, *P*=0.13, MRSA, *P*=0.14). There was no significant difference between urban and rural children for ESCrE (47.7%, 74/155 and 53.4%, 135/253, *P*=0.31); both CRE and MRSA were rarely detected (< 2%) with no difference across settings (CRE, *P*=1.0, MRSA, *P*=0.42). Among Enterobacteriaceae recovered, *Escherichia coli* and *Klebsiella* spp. predominated.

**Conclusion:**

Colonization with AROs were widespread in households and hospitals in urban and rural areas. Hospitals with elevated prevalence of highly transmissible AROs should consider whether implementation of colonization screening can be incorporated as part of their infection prevention and control programs. Risk factors for ARO colonization should be elucidated to identify novel prevention strategies.

**Disclosures:**

**All Authors**: No reported disclosures

